# Elicitation of expert prior opinion: application to the mypan trial in childhood polyarteritis nodosa

**DOI:** 10.1186/1546-0096-12-S1-P125

**Published:** 2014-09-17

**Authors:** Lisa Hampson, John Whitehead, Despina Eleftheriou, Catherine Tudur-Smith, Rachel Jones, David Jayne, Helen Hickey, Paul Brogan

**Affiliations:** 1 Department of Mathematics and Statistics, Lancaster University, Lancaster, UK; 2 Paediatric Rheumatology, Institute of Child Health, London, UK; 3 MRC North West Hub for Trials Methodology Research, Department of Biostatistics, Liverpool University, Liverpool, UK; 4 Department of Renal Medicine, Addenbrookes Hospital, Cambridge, UK; 5 Medicines for Children Research Network Clinical Trials Unit, University of Liverpool, Liverpool, UK

## Introduction

A major challenge in rare diseases is conducting clinical trials with sufficient power to inform best clinical practice when anticipated sample sizes are small. Historically, this has been a major barrier in rare paediatric autoimmune diseases. Bayesian methodology can be used to augment the sparse therapeutic data obtained from clinical trials in these circumstances.

## Objectives

We elicited expert prior opinion for a future Bayesian randomised controlled trial for a rare inflammatory paediatric disease, polyarteritis nodosa (MYPAN, Mycophenolate mofetil for polyarteritis nodosa).

## Methods

A Bayesian prior elicitation meeting was convened. Participating experts were drawn from across the EU and Turkey. Opinion was sought on the probability that a patient in the MYPAN trial treated with cyclophosphamide would achieve disease remission within 6-months, and on the relative efficacies of mycophenolate mofetil and cyclophosphamide. Expert opinion was combined with previously unseen data from a recently completed randomised controlled trial of mycophenolate mofetil versus cyclophosphamide in anti-neutrophil cytoplasmic antibody associated vasculitis.

## Results

A pan-European group of fifteen experts participated in the elicitation meeting. Consensus expert prior opinion was that the most likely rates of disease remission within 6 months on cyclophosphamide or mycophenolate mofetil were 74% and 71% respectively. This prior opinion will now be taken in to account and will be modified to formulate a Bayesian posterior opinion when data from 40 patients completing the trial randomised at a 1:1 ratio to either receive cyclophosphamide or mycophenolate mofetil are available.

## Conclusion

We suggest that this methodological template could be applied to trial design for other rare diseases, and is of particular relevance to rare autoimmune conditions that currently lack a good evidence base for treatment.

## Disclosure of interest

None declared.

